# Examining the Characteristics of Adolescents Recruited to a Novel Digital Treatment for Eating Disorders: Baseline Assessment in an Open Feasibility Trial

**DOI:** 10.2196/93431

**Published:** 2026-08-21

**Authors:** Guri Holgersen, Emilie Sektnan Nordby, Irene Bircow Elgen, Ester Marie Stornes Espeset, Tine Nordgreen

**Affiliations:** 1 Department of Global Public Health and Primary Care, Faculty of Medicine, University of Bergen, P.O. Box 7800, Bergen, Vestland, 5020, Norway, 47 90737908; 2 Division of Psychiatry, Haukeland University Hospital, Bergen, Vestland, Norway; 3 Department of Child and Adolescent Psychiatry, Haukeland University Hospital, Bergen, Vestland, Norway; 4 Department of Clinical Medicine, Faculty of Medicine, University of Bergen, Bergen, Vestland, Norway; 5 Department of Child and Adolescent Psychiatry, Helse Fonna, Haugesund, Norway

**Keywords:** eating disorders, adolescents, digital treatment, routine clinical care, developing novel intervention, characteristics of help-seeking

## Abstract

**Background:**

Digital interventions for eating disorders have the potential to increase access to care and broaden the range of treatment options for adolescents. However, little is known about the characteristics of those who seek these interventions. Identifying such characteristics will help ensure that these interventions meet the needs of the target population, highlight potentially underrepresented groups, and support clinicians in assessing clinical suitability for individual patients.

**Objective:**

This study aimed to examine the characteristics of adolescents seeking digital treatment for eating disorders within routine clinical care and to explore whether the key components of the novel digital treatment align with the characteristics of the adolescents it is designed to support.

**Methods:**

This study used baseline data from an open feasibility trial of a novel digital treatment for eating disorders within routine clinical care. Participants were adolescents aged 15 to 18 years with a diagnosis of atypical anorexia nervosa, atypical bulimia nervosa, binge-eating disorder, or eating disorder, unspecified. Baseline assessment included demographic characteristics, eating disorder symptomatology, psychosocial impairment, emotional dysregulation, anxiety, depression, negative self-evaluation, and motivational factors.

**Results:**

A total of 25 adolescents participated in the study, all of whom were women. The mean age was 16 (SD 0.87) years. Half of the adolescents had previously received face-to-face treatment for an eating disorder. The sample was transdiagnostic with some variation in distribution. The adolescents reported severe eating disorder symptoms, marked psychosocial impairment, emotional difficulties, elevated negative self-evaluation, and high levels of internal pretreatment motivation.

**Conclusions:**

The findings from this study underscore the relevance of digital interventions to complement or extend traditional eating disorder care for adolescents. This study emphasizes the importance of designing digital treatments that are sensitive to normative biases, address the multifaceted nature of eating disorders, and are tailored to the needs and preferences of a transdiagnostic population. The present findings are important insofar as they may inform whether the key treatment components align with the characteristics of adolescents seeking digital treatment for eating disorders within routine clinical care.

## Introduction

Eating disorders are a group of complex clinical conditions characterized by pathological concerns about shape and weight, as well as disturbed eating and weight-control behaviors [1]. Impaired physical health, disrupted psychosocial functioning, and reduced life expectancy are some of the severe consequences of eating disorders [2,3]. The disorders are highly prevalent worldwide, especially in adolescents [4,5], with overall estimates suggesting that approximately 1 in every 20 children and adolescents are affected [6]. Several evidence-based treatments are available for adolescents [7], with family-based treatment and enhanced cognitive behavioral therapy having the strongest evidence base [8].

Despite the severity of eating disorders and the availability of evidence-based treatments, most individuals who meet the criteria for a clinical eating disorder do not seek help [9,10]. Eating disorders are therefore frequently undetected and untreated [3,11], with particularly low help-seeking rates among adolescents, where only 10% to 20% access treatment [12-14]. Individual barriers to help-seeking behavior include poor mental health literacy, stigma surrounding eating disorders, denial of the disorder’s severity, as well as distorted body ideals enforced by social media [9,15]. Systemic factors contributing to the existing treatment gap are limited therapist availability, the cost of treatment, geographic isolation from available services, long waitlists, and poor eating disorder literacy among primary care providers [9,16]. Moreover, of those who seek help, a substantial number do not respond to the treatments currently available [17,18]. High dropout rates (29%‐73%) [19,20] and low remission rates (40%‐50%) indicate that a substantial proportion continue to exhibit high levels of eating pathology at the end of treatment [17,21]. Additional factors have been identified as contributing to the complexity of treating eating disorders. The disorders’ complex interplay of psychological, biological, and social factors [15,22], the underlying mechanisms being unknown [23], and the high psychiatric comorbidity (>70%) [24] could be among the reasons. Furthermore, eating disorder treatments often have a categorical approach, despite diagnostic migration being common and diagnoses varying across individuals [25]. In addition, treatments often follow standardized protocols and manuals, which do not account for unique individual factors influencing an eating disorder [26]. As a result, individuals with eating disorders can feel alienated from treatment [27,28]. Given the aforementioned challenges in treating eating disorders, there is a pressing need to both improve access to existing evidence-based treatments and develop novel interventions that address the multifaceted nature of these conditions [17,23,26,29].

A promising approach is the use of treatments delivered via computers or smartphones, often referred to as digital interventions or digital treatments [30,31]. The accessibility of these treatments positions them as a valuable component within the spectrum of services for eating disorders, particularly considering the numerous barriers associated with traditional treatments [32]. Digital interventions can increase access to care and have demonstrated effectiveness in improving symptoms of eating disorders while also being cost-effective [7,33]. While endorsing many of these advantages, most individuals with eating disorders still prefer face-to-face treatment [34]. Moreover, despite a growing evidence base, only a limited number of digital interventions have been specifically developed for adolescents with eating disorders [31]. At present, the few available app-based interventions, such as TCApp and Recovery Record, have primarily been evaluated as adjuncts to standard treatment rather than as standalone interventions [35,36]. In addition, digital interventions also face challenges related to uptake and engagement [30,37], and one contributing factor may be expert-driven development processes that do not sufficiently reflect the preferences and goals of the intended users [38,39].

To address these challenges, there is a need to develop novel digital interventions for adolescents with eating disorders [17,31] based on the perspectives of those who will use them [37,40]. This is particularly important given that adolescents differ from adults in their developmental needs, preferences, and contexts, making simple adaptations of adult interventions insufficient [41,42]. At the same time, digital interventions may not be acceptable or suitable for all individuals, underscoring the importance of understanding which adolescents engage with such approaches and who may benefit most [34,37]. Such knowledge can also help identify underrepresented groups and inform necessary adaptations to ensure that interventions fit into adolescents’ everyday lives [37,43]. The involvement of adolescents in the development of such interventions remains relatively limited, raising concerns about whether existing tools adequately reflect their needs and preferences [44,45]. Furthermore, only a small number of digital interventions have been developed and evaluated within routine clinical care, where treatments are delivered under real-world conditions with heterogeneous patient populations and varying clinical practices [16,38,46]. As a result, it remains unclear which adolescents are reached by digital interventions in these settings and whether those who engage with them correspond to the intended target population. Addressing this gap is essential for evaluating the feasibility and clinical relevance of digital treatments. Assessing sample representativeness within feasibility trials can provide important insights into whether interventions reach the populations they are designed for and help identify potential barriers to implementation. This study therefore aimed, as part of a feasibility trial of a novel digital treatment, to examine the characteristics of adolescents seeking digital treatment for eating disorders within routine clinical care and to explore whether the key components of the novel digital treatment align with the characteristics of the adolescents it is designed to support.

## Methods

### Study Design

An open feasibility trial of a digital treatment for adolescents with eating disorders was conducted in routine clinical care. The trial was guided by the UK Medical Research Council’s framework for developing and evaluating complex interventions and followed CONSORT (Consolidated Standards of Reporting Trials) standards for feasibility trials (Checklist 1) [47,48]. In line with these recommendations, 4 key progression criteria were assessed to evaluate feasibility across multiple domains: sample representativeness, adherence, acceptability, and preliminary efficacy. In this study, we specifically report findings related to sample representativeness as an indicator of feasibility. Sample representativeness was examined in relation to overall eating disorder psychopathology to better understand which adolescents sought digital treatment and the extent to which the participants reflected the intended target population. This was assessed by analyzing baseline characteristics from the feasibility trial and evaluating how well the key components of the intervention corresponded to the clinical profile of the sample. The study used baseline data from participants included in the feasibility trial between March 2024 and May 2025. In accordance with feasibility trial design, no formal sample size calculation was conducted.

### Study Setting

Adolescents receiving the digital treatment were patients at child and adolescent psychiatric outpatient clinics in Norway, specifically within the catchment area of Haukeland University Hospital and Helse Fonna. These clinics are part of the state-funded Norwegian specialist health care system and are located within the Western Norway Regional Health Authority, serving a population of approximately 122,000 youth under 18 years [49].

### Study Population

#### Inclusion Criteria

The following inclusion criteria were applied for the feasibility trial: (1) diagnosed with atypical anorexia nervosa, atypical bulimia nervosa, binge-eating disorder, or eating disorder, unspecified [50,51]; (2) aged between 15 and 18 years; (3) on a stable dose of medication for a psychiatric disorder for 6 weeks; (4) has a mobile phone with internet access; and (5) speaks and writes Norwegian.

#### Exclusion Criteria

The following exclusion criteria were applied: (1) diagnosed with anorexia nervosa, bulimia nervosa, or avoidant or restrictive food intake disorder (ARFID); (2) having a comorbid medical condition or disorder known to influence eating or weight (ie, pregnancy, cancer); (3) psychotic disorders, acute suicidality, substance abuse, substance dependence, or a severe depressive episode; and (4) receiving inpatient treatment for a psychiatric disorder or face-to-face psychological treatment. This trial was the first to examine a digital intervention for adolescents with eating disorders within routine clinical care in Norway. During the planning phase, concerns were raised about including participants with anorexia nervosa or bulimia nervosa due to the increased risk of medical instability. Although a more flexible inclusion strategy based on clinician judgment was considered, it was not implemented due to concerns about maintaining internal validity. As a result, adolescents with anorexia nervosa and bulimia nervosa were excluded from the trial. Patients with ARFID were excluded since the clinical picture is different from that of other eating disorders [50].

### Procedure

Participants were recruited from 8 child and adolescent psychiatric outpatient clinics. Information about the study was disseminated through meetings with clinic managers, emails to staff, and flyers placed in waiting rooms. Interested participants were provided with a link to the study website for information about the study and a brief preliminary online screening. The online screening captured information on disordered eating, eating-related behaviors, functional impairment in daily life, as well as key inclusion criteria such as age, internet access, and language. Eligible participants were given the opportunity to leave their contact information and were contacted by telephone to confirm inclusion and exclusion criteria. A diagnostic assessment was conducted by trained clinicians within the research group using the Mini-International Neuropsychiatric Interview (MINI) [52] and Eating Disorder Assessment for *DSM-5* (EDA-5) [53]. As the *International Statistical Classification of Diseases, Tenth Revision* (*ICD-10)* is the standard diagnostic framework in Norway, final diagnoses and all inclusion and exclusion criteria were determined according to *ICD-10* [51]. While MINI was used as a diagnostic instrument, the EDA-5 served to systematically assess eating disorder symptoms and support clinical judgment, rather than as an independent diagnostic tool. In response to slow recruitment, additional promotion of the study was carried out via upper secondary schools and advertisements on social media. As the study was conducted in routine clinical care, all participants had to be eligible for specialized care in accordance with national priority guidelines [54]. Participants who were not already patients at an outpatient clinic were required to visit their general practitioner to confirm medical stability and obtain a referral. Eligible participants were given access to the native treatment application, in which they signed consent forms, with those aged 15 years required to provide additional parental consent.

### Intervention

**Figure 1.**
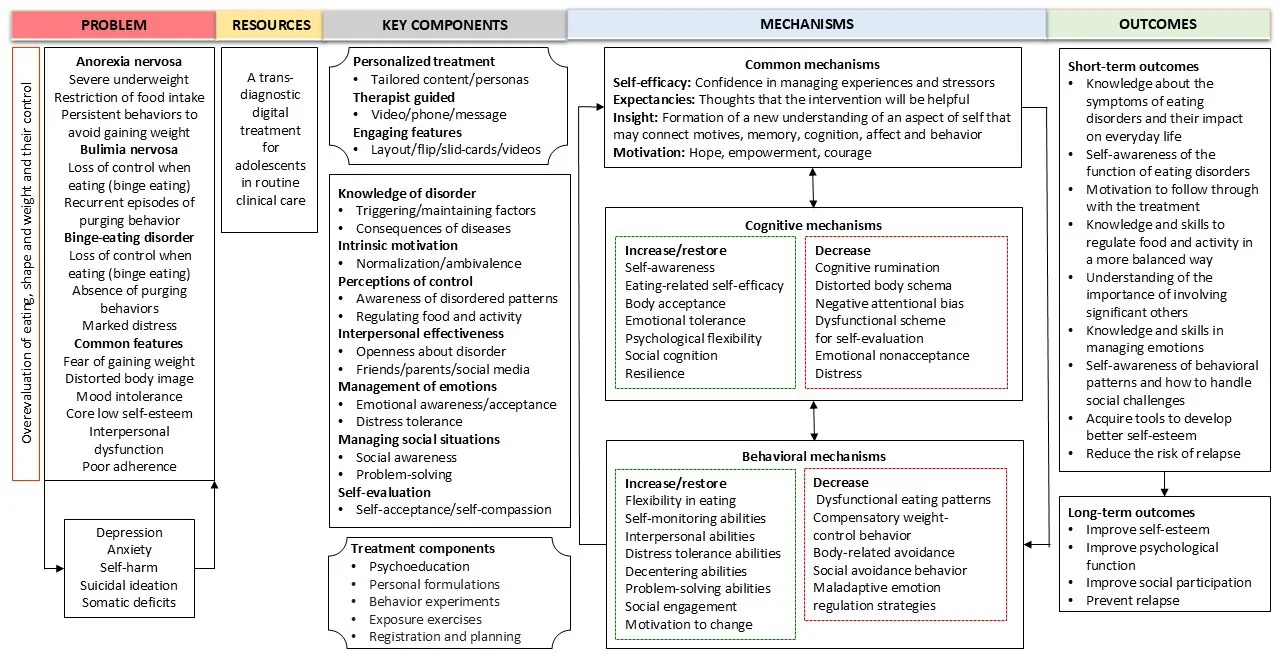
Logic model.

The intervention was developed in line with the person-based approach (PBA) [55]. PBA is a methodological framework for understanding the most effective way to apply appropriate behavior change techniques in the specific context of the intervention and its intended users [55]. In line with PBA, adolescents with lived experience of eating disorders [27], mental health professionals, and the existing evidence base helped us identify key issues, needs, and challenges the intervention should address. Drawing on this in-depth understanding, key components were identified and systematically embedded into the design of the intervention. A logic model was created to describe how the key components of the intervention should lead to positive behavior change and better health (Figure 1). During development, the key components were incorporated and user-tested to refine the digital treatment to meet user requirements. This model serves as background for the second aim of this study: exploring whether the following key components of the novel digital treatment align with the adolescents it is designed to support: eating disorder symptomatology, psychosocial impairment, emotional difficulties (emotion dysregulation, anxiety, and depression), negative self-evaluation, and motivational factors.

The development process resulted in a therapist-guided digital treatment delivered through a smartphone app. The intervention is based on a transdiagnostic cognitive behavioral framework targeting mechanisms that maintain eating disorders, alongside elements from emotion regulation approaches to enhance distress tolerance [56,57]. The treatment consists of eight modules delivered in a fixed sequence: (1) What is an eating disorder? (2) Motivation and change, (3) Balancing food and activity, (4) Involving others, (5) Management of emotions, (6) Managing social situations, (7) Improving self-esteem, and (8) Preventing relapse. Modules were released weekly, although 2 were designed to be completed over a 2-week period (modules 3 and 5). Adolescents received weekly clinician support through scheduled telephone consultations lasting approximately 15 to 30 minutes. In addition, they could send asynchronous messages to clinicians to address technical or practical issues. During the feasibility trial, the intervention was incorporated into the existing workflows of participating Child and Adolescent Mental Health Services (CAMHS) outpatient clinics. The clinicians delivering the intervention were employed within CAMHS and were assigned approximately 10% of a full-time position, corresponding to a caseload of around 3 patients each. Standardized guidelines were developed to support clinicians in delivering the intervention. Clinicians also received brief introductory training on the digital platform and treatment procedures. In addition, they participated in fortnightly 1-hour group supervision sessions led by an experienced clinical psychologist.

### Measures

Data were collected using self-report questionnaires administered online. Sociodemographic data (age, gender, living situation, engagement at school and with friends) were gathered using a questionnaire developed specifically for this study. The selected measures have been applied in adolescent and eating disorder populations, used in evaluations of digital interventions for this group, and are consistent with international consensus recommendations on patient-centered outcome measures in eating disorders [58,59].

#### Eating Disorder Examination—Questionnaire Short (EDE-QS)

Eating disorder symptoms were measured using the EDE-QS [60]. EDE-QS is a 12-item questionnaire with a 4-point response scale that assesses symptom severity over the preceding 7 days. Scores range from 0 to 36, and higher scores indicate greater symptom severity. A score of 15 or above serves as a cutoff point for distinguishing between eating disorder cases and noncases [61]. The questionnaire has high internal consistency (Cronbach α=0.91) [60].

#### Clinical Impairment Assessment Questionnaire (CIA)

CIA was used to assess the severity of psychosocial impairment due to eating disorder features [62]. CIA consists of 16 items covering impairment in different domains of life that are typically affected by eating disorder psychopathology. Three subscales are computed, representing personal, social, and cognitive impairment that can result from eating disorders. Global scores range from 0 to 48, with higher ratings indicating a higher level of impairment [62]. A global score of 16 or above represents a cutoff for clinically significant impairment [62]. Normative data from a clinical adult female sample in Norway indicate mean scores of 14.01 (SD 4.31) for the personal impairment subscale, 9.54 (SD 3.97) for the social impairment subscale, and 8.97 (SD 3.81) for the cognitive impairment subscale [63]. In this study, the normative data have been rounded to the nearest whole number to report the number of participants with a mean score that matches the clinical sample. The questionnaire has high internal consistency (Cronbach α=0.97) [62].

#### Difficulties in Emotion Regulation Scale (DERS-18)

The DERS-18 was used to assess clinically relevant difficulties in emotion regulation [64]. Participants answer on a 5-point Likert-type scale ranging from 1 (“almost never”) to 5 (“almost always”). Scores range from 18 to 90, with higher scores indicating greater emotion dysregulation [64]. DERS-18 consists of 6 subscales measuring difficulties in the flexible, multidimensional regulation of emotion: lack of awareness of one’s emotions (awareness), lack of acceptance of one’s emotions (nonacceptance), lack of access to effective emotion regulation strategies (strategies), lack of ability to manage one’s impulses during negative emotions (impulse), lack of ability to engage in goal-directed activities during negative emotions (goals), and lack of clarity about the nature of one’s emotions (clarity) [64]. DERS-18’s internal consistency is very high (Cronbach α=0.90) [65]. DERS is ideal for use in clinical research studies that require multiple assessment points and is frequently used in treatment outcome research [65]. Due to the different versions of the DERS, it is difficult to collapse findings across studies [65]. In one study from 2022, DERS scores were organized by depression subgroups [66]. For the DERS-18, total mean scores were 35.88 (SD 8.69) for normal subgroup, 42.68 (SD 10.50) for mild depression, 48.39 (SD 10.15) for moderate depression, and 56.13 (SD 10.08) for severe depression [66]. In this study, we used the same putative depressive symptomatology subgroups to organize our sample’s difficulties with emotion regulation. We used the moderate depression subgroup as a cutoff for difficulties within each domain. The reported cutoff scores have been rounded to the nearest whole number.

#### Generalized Anxiety Disorder-2 Scale (GAD-2)

Anxiety symptoms were measured using the 2-item version of the GAD-2 [67]. GAD-2 measures symptoms over the last 2 weeks, scored on a 4-point Likert scale ranging from 0 (“not at all”) to 3 (“nearly every day”), with a cutoff score of 3 or above [68].

#### Patient Health Questionnaire-2 (PHQ-2)

Symptoms of depression were measured using the 2-item version of the PHQ-2 [69]. PHQ-2 measures symptoms over the last 2 weeks and is scored on a 4-point Likert scale ranging from 0 (“not at all”) to 3 (“nearly every day”) [70]. The standard cutoff score of PHQ-2 is 3 or above; however, a cutoff score of 2 or above may represent an optimal threshold for adolescents [71].

#### Rosenberg Self-Esteem Scale (RSES)

The RSES was used to measure negative self-evaluation [72]. RSES measures self-competence and self-liking using 10 items, answered on a 4-point Likert-type scale—from “strongly agree” to “strongly disagree.” The scale ranges from 0 to 30, and scores between 15 and 25 are within the normal range; scores below 15 suggest low self-esteem [72]. The questionnaire’s Cronbach α was 0.92, indicating excellent internal consistency [72].

#### Total Burden of Symptoms

To capture the overall level of symptom burden among participants, a composite score ranging from 0 to 6 was calculated. For each standardized measure, 1 point was assigned if the participant’s score exceeded the established clinical cutoff. The total score therefore reflects the number of measures for which the participant scored above the cutoff, with higher scores indicating a greater overall symptom burden.

#### Treatment Motivation

In addition to the standardized measures, the participants were given a 6-item questionnaire to explore motivational factors for participating in a novel treatment. As no suitable validated instrument was available, the questionnaire was developed specifically for this study, drawing on the Short Motivation Feedback List [73] and grounded in self-determination theory [74]. Four items were rated on a 5-point Likert scale ranging from “Strongly disagree” to “Strongly agree” and were designed to capture both the level and type of motivation (internal vs external). These items assessed reasons for participating in treatment, including intrinsic motivation (eg, wanting to engage in treatment) and externally driven motives (eg, perceived expectations from others). The remaining 2 items asked participants to rate, on a scale from 0 to 100, how much effort they expected to invest in the treatment and the extent to which they would involve others for support when needed.

### Data Analysis

Data were analyzed using SPSS (version 29). Given the descriptive nature of this study, data are presented in terms of frequencies, percentages, means, and standard deviations.

### Ethical Considerations

This study was approved by the Regional Committees for Medical and Health Research Ethics in Norway (REC-639031). In addition, the study was conducted in line with the Haukeland University Hospital’s guidelines for ethics and privacy considerations. Informed consent was obtained via the smartphone app.

## Results

### Recruitment

**Figure 2.**
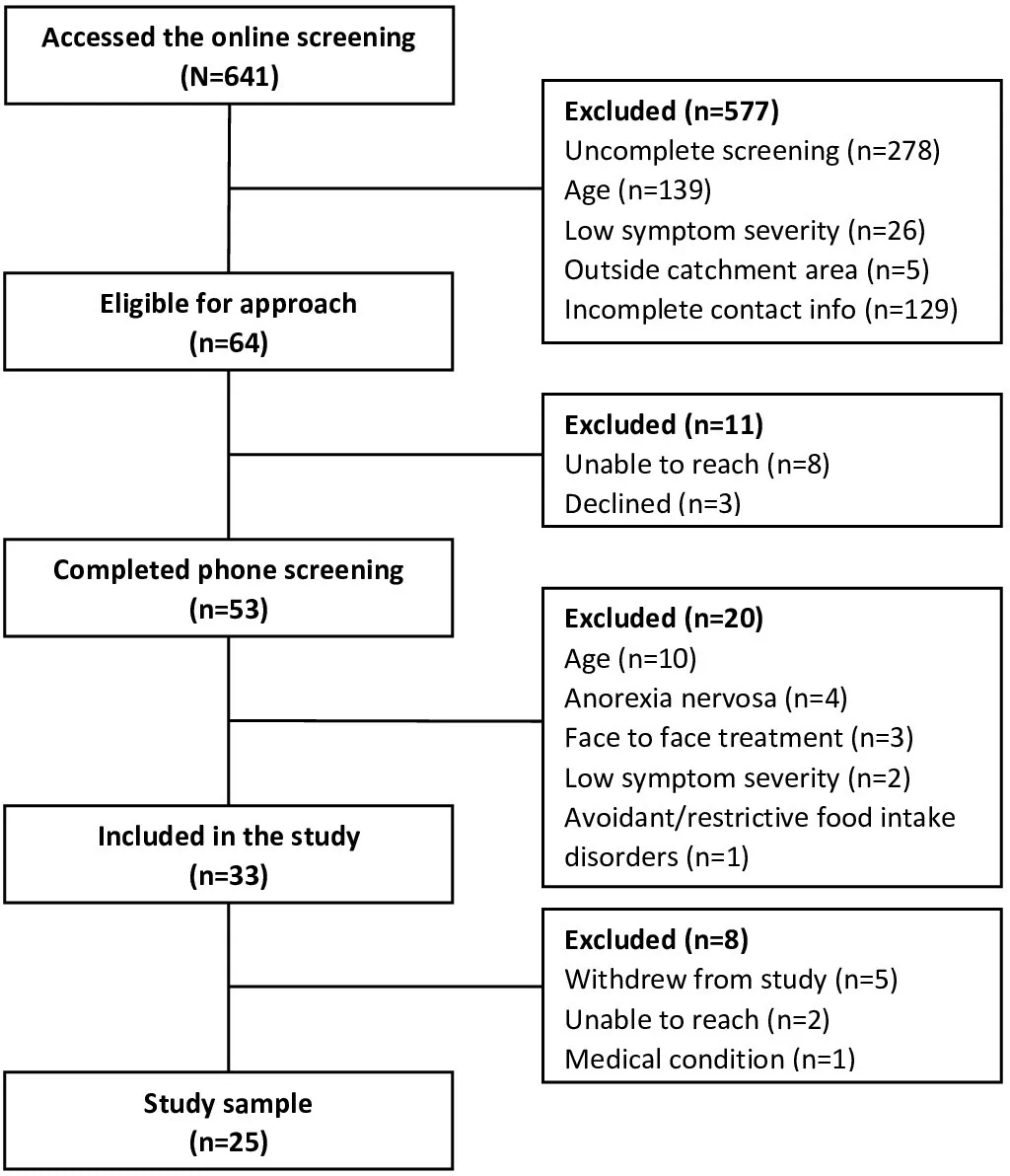
Recruitment flowchart.

Following 14 months of recruitment, baseline data were used for this study as the project neared completion. A total of 641 individuals accessed the online screening portal (Figure 2) between March 2024 and May 2025. Of these, 577 individuals were ineligible due to incomplete screening, being outside the age range, not meeting diagnostic criteria for an eating disorder (low symptom severity), or living outside the catchment area. In addition, 129 individuals met the criteria for the online screening but could not be contacted as they did not leave their contact information. Of the 64 adolescents eligible for approach, 11 were excluded because they were unable to be reached or declined participation. Of the 53 adolescents contacted by telephone, 20 were excluded from the study due to age (n=10), a primary diagnosis of anorexia nervosa (n=4), receiving face-to-face psychological treatment (n=3), low symptom severity (n=2), or ARFID (n=1). After inclusion, 5 adolescents changed their minds regarding participation, 2 were unable to be reached, and 1 was excluded due to a medical condition. Twenty-five adolescents completed the baseline screening and enrolled in the study. These were recruited through CAMHS (n=15), upper secondary schools (n=5), and social media (n=5).

### Sociodemographic Characteristics

All participants were women (Table 1). The mean age of the study sample was 16 (SD 0.87, range 15-18) years. Over half of the sample were living with both parents (60%). All participants were engaged in school (100%), and almost all (96%) were engaged with friends on a regular basis.

**Table 1.** Sociodemographic characteristics of adolescents seeking digital treatment (N=25).

Characteristics	Values
Gender, n (%)	
Men	0 (0)
Women	25 (100)
Nonbinary	0 (0)
Age (y), mean (SD)	16.48 (0.87)
Living situation, n (%)	
Both parents	15 (60)
50/50 residential	3 (12)
One parent	4 (16)
Alone	1 (4)
Other living agreement	2 (8)
Engaged in education, n (%)	
Reduced time	5 (20)
Full-time	20 (80)
Engaged with friends^a^, n (%)	
Daily	6 (24)
Weekly	11 (44)
Monthly	7 (28)

a For the sociodemographic characteristic “Engaged with friends,” N=24 (96%).

### Diagnoses and History of Present Illness

Most of the adolescents were diagnosed at inclusion with atypical anorexia nervosa (40%), with the second largest group being eating disorder, unspecified (28%) (Table 2). The rest of the sample was equally distributed between atypical bulimia nervosa (16%) and binge-eating disorder (16%). The mean age at eating disorder onset was 13 (SD 1.68, range 9‐16) years. Half of the adolescents had previously received face-to-face treatment for an eating disorder (52%, 13/25).

**Table 2.** Diagnoses and history of present illness of adolescents seeking digital treatment (N=25).

Characteristics	Values
Diagnoses (inclusion), n (%)	
Atypical anorexia nervosa	10 (40)
Atypical bulimia nervosa	4 (16)
Binge eating disorder	4 (16)
Eating disorder, unspecified	7 (28)
Prior eating disorder treatment, n (%)	
Yes	13 (52)
No	12 (48)
Age of disorder onset (y), mean (SD)	13.00 (1.68)

### Motivational Factors

The adolescents’ motivational factors for participating in a novel digital treatment are summarized in Table 3. Most of the adolescents’ responses to items 1 and 3 were in the higher categories, 4 (“agree”) and 5 (“strongly agree”), indicating high internal treatment motivation. When asked to rank (0%‐100%) how much effort they would put into carrying out the treatment, the mean score was 83% (SD 14.34%), ranging from 50% to 100%. Regarding the involvement of others, the mean score was somewhat lower (52%, SD 33.18%), with a range from 0% to 100%.

**Table 3.** Motivational factors among adolescents seeking digital treatment (N=25).

Item number	Items	Median (IQR)	n (%)	n (%)	n (%)	n (%)	n (%)
	How much do you agree with the following statements:		Strongly disagree	Disagree	Neither disagree nor agree	Agree	Strongly agree
1	I am participating in treatment because I want to.	5 (4-5)	1 (4)	0 (0)	1 (4)	10 (40)	13 (52)
2	I am participating in treatment because others think I should.	3 (3-4)	1 (4)	4 (16)	8 (32)	9 (36)	3 (12)
3	I am participating in treatment because it will help me live a better life.	4 (4-5)	2 (8)	0 (0)	1 (4)	12 (48)	10 (40)
4	I am participating in treatment to avoid disappointing others.	2 (2-3.5)	5 (20)	9 (36)	5 (20)	5 (20)	1 (4)

### Eating Disorder Symptoms

The distribution of eating disorder symptoms was in the severe range, with a mean EDE-QS score of 21.56 (SD 6.80) [61]. Table 4 provides an overview of the distribution of symptoms, range, cutoff, and mean.

**Table 4.** Distribution of eating disorder symptoms, psychosocial impairment, emotional difficulties, and negative self-evaluation (N=25).

Measures	Range	Cutoff^a^, n (%)	Mean (SD)
Eating Disorder Examination—Questionnaire Short (EDE-QS)			
Total score	8-33	21 (84)	21.56 (6.80)
Clinical Impairment Assessment Questionnaire (CIA)			
Global score	8-47	22 (88)	28.08 (10.71)
Personal impairment	5-18	3 (52)	13.08 (3.89)
Social impairment	1-15	9 (36)	7.80 (4.10)
Cognitive impairment	2-14	10 (40)	7.20 (3.52)
Difficulties in Emotion Regulation Scale-18 (DERS-18)			
Total score	32-85	17 (68)	53.72 (13.92)
Awareness	4-13	21 (84)	10.00 (2.48)
Nonacceptance	3-15	18 (72)	8.92 (3.39)
Strategies	3-15	11 (44)	7.24 (3.64)
Impulse	3-15	15 (60)	7.52 (3.68)
Goals	5-15	15 (60)	11.16 (2.88)
Clarity	3-15	15 (60)	8.88 (2.86)
Generalized Anxiety Disorder-2 Scale (GAD-2)			
Total score	1-6	17 (68)	3.44 (1.69)
Patient Health Questionnaire-2 (PHQ-2)			
Total score	0-6	20 (80)	2.60 (1.56)
Rosenberg Self-Esteem Scale (RSES)			
Total score	0-28	19 (76)	11.76 (6.33)

a Participants with scores exceeding the clinical cutoff on measure were identified. For the CIA subscales and the DERS-18, normative data were used due to the lack of clinically validated cutoff scores (62 and 66).

### Psychosocial Impairment

The global impairment score was high at 28.08 (CIA) [62] (Table 4). The impairment subscales show difficulties across the personal, social, and cognitive domains [63].

### Emotional Difficulties

To explore the adolescents’ emotional difficulties, emotion dysregulation and symptoms of anxiety and depression were assessed (Table 4). The sample’s abilities to regulate emotions indicate moderate to severe difficulties within this domain, with a total mean DERS-18 score of 53.72 (SD 13.92) [66]. The adolescents had difficulties in all the 6 DERS-18 subscales. In addition, they reported anxiety symptoms above the cutoff (GAD-2 score 3.44, SD 1.69) [68]. The mean score for depression was below the cutoff (PHQ-2 score 2.60, SD 1.56) [70]; however, using an optimal cutoff score for adolescents of 2 or above, the score exceeded the threshold [71].

### Negative Self-Evaluation

To evaluate the sample’s level of negative self-evaluation, we measured the adolescents’ self-esteem (RSES mean score 11.76, SD 6.33), which was in the range indicating severely low self-esteem [72].

### Total Burden of Symptoms

**Figure 3.**
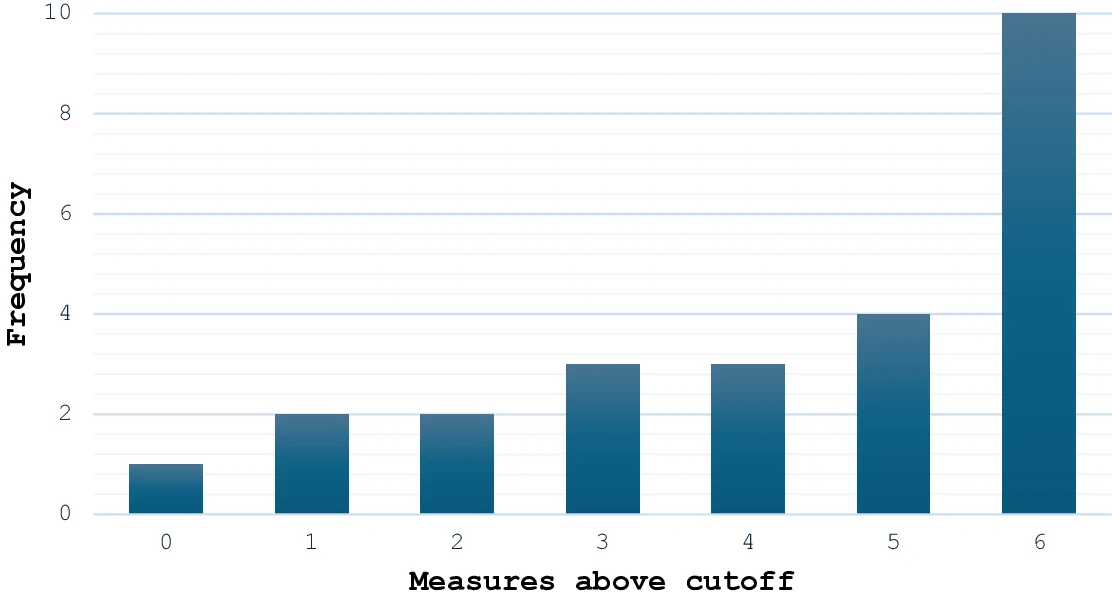
Total burden of symptoms above established cutoffs among adolescents seeking digital treatment (N=25). Each standardized measure exceeding its clinical cutoff contributes one point to the total symptom burden score. A score of 1 indicates one measure above cutoff, and a score of 2 indicates 2 measures above cutoff, and so forth.

The total burden of symptoms was calculated (Figure 3). Median response scores ranged from 0 to 6, with the median score on 5 measures above the cutoff.

### Alignment Between Key Components and Adolescents’ Characteristics

The second aim of this study was to explore whether the key components of the novel intervention (for more details, see Figure 1) align with the characteristics of the adolescents recruited for this study. Table 5 shows an overview of the key components, the results from measuring these characteristics, and an evaluation of the alignment.

**Table 5.** Alignment between key components of the digital treatment and adolescents’ characteristics.

Key components	Measure	Result	Evaluation	Alignment
Eating disorder symptomatology	EDE-QS^a^	Majority above clinical cutoff	Indicates that the treatment targets core symptoms relevant to the adolescent population.	Yes
Impairment	CIA^b^	High level of global impairment	Suggests that the intervention’s focus on functioning is well-matched with the sample’s needs.	Yes
Emotional difficulties	DERS-18^c^ GAD-2^d^ PHQ-2^e^	High levels of anxiety, depression, and emotional dysregulation	Supports the relevance of the treatment in addressing emotional difficulties across all these domains.	Yes
Negative self-evaluation	RSES^f^	Severe low self-esteem	Confirms the importance of components addressing self-image and self-worth.	Yes
Motivational factors	Six-item questionnaire	High level of pretreatment motivation	Levels observed were higher than anticipated. Variability across individuals suggests that tailored engagement strategies may still be necessary to optimize uptake and adherence.	Partial

a EDE-QS: Eating Disorder Examination—Questionnaire Short.

b CIA: Clinical Impairment Assessment Questionnaire.

c DERS-18: Difficulties in Emotion Regulation Scale-18.

d GAD-2: Generalized Anxiety Disorder-2 Scale.

e PHQ-2: Patient Health Questionnaire-2.

f RSES: Rosenberg Self-Esteem Scale.

## Discussion

### Principal Findings

As part of examining the feasibility of a novel digital treatment, this study assessed sample representativeness. Specifically, the study examined the characteristics of adolescents seeking digital treatment for eating disorders within routine clinical care and explored whether the key components of the novel digital treatment aligned with the characteristics of the adolescents it is designed to support. Although recruitment was not specified as a predefined feasibility criterion, the findings revealed substantial challenges in this area. Given the central role of recruitment in feasibility research, particularly for understanding whether the intended population can be reached, these challenges are addressed first in the discussion to provide important context for interpreting the remaining findings.

### Recruitment

The results revealed considerable challenges in enrolling participants. Despite substantial traffic to the online screening portal, only a small proportion of adolescents progressed to study participation, suggesting a gap between initial interest and actual engagement. This pattern may reflect well-established features of eating disorders, such as ambivalence toward treatment and fluctuating motivation, which can reduce readiness to seek or accept help [3,9]. Additional challenges may be related to adolescence as a developmental stage, where factors such as stigma, reliance on caregivers, and competing demands can influence help-seeking behavior [75]. Intervention-specific and contextual factors may also have contributed. Some adolescents may have been hesitant to engage in a digital treatment format or may have preferred traditional face-to-face care [34,37]. At the same time, recruitment via social media appeared to reach individuals who had not previously accessed clinical services, indicating an unmet need for more accessible entry points to care. It should also be noted that recruitment challenges are common in clinical research [76]. In this study, such challenges may have been further exacerbated by practical constraints within clinical settings, including limited time and resources among clinicians. Together, these findings suggest that while digital interventions may lower initial barriers to help-seeking, they do not fully overcome challenges related to participation in studies and treatment.

### Characteristics of Adolescents Seeking Digital Treatment for Eating Disorders

Atypical anorexia nervosa was the most frequently assigned diagnosis, which aligns with data from a Norwegian community-based prevalence study [10]. Furthermore, the adolescents’ distributions of eating disorder symptoms and psychosocial impairment are consistent with the diagnostic thresholds for inclusion in this study. Moreover, their elevated levels of emotional difficulties and negative self-evaluation are in accordance with other studies examining these mechanisms among adolescents with eating disorders [77-79]. Low self-esteem and mood intolerance are particularly pertinent in the adolescent age range [78], and anxiety and depression are the most prevalent comorbid disorders [79]. The adolescents’ total burden of symptoms across domains emphasizes the sample’s severity. The adolescents reported a high level of internal pretreatment motivation. The strong internal motivation may be linked to the adolescents’ severe eating disorder symptoms [9] or to their being more likely to engage in the intervention due to recruitment via a self-selection process [80]. Providing adolescents with a sense of control and agency in treatment decision-making may contribute to improved adherence to therapeutic interventions [37].

The fact that none of the adolescents included in this study were men reflects the well-established gender discrepancies in the field of eating disorders [5]. Stigma associated with the disclosure of mental health issues and cultural perceptions that eating disorders are typically female disorders might influence help-seeking behaviors among adolescent boys [81]. Moreover, although individuals that identify as transgender or nonbinary are at particularly high risk for developing eating disorders [82,83], no participants in this study identified with a gender other than women. The results of this study highlight the presence of underrepresented groups within the adolescent population seeking treatment for eating disorders [17,81,83].

### Alignment of Novel Interventions With Adolescents’ Characteristics

The distribution of eating disorder symptoms within the sample indicates that the treatment targets core symptoms relevant to the population. In addition, the intervention’s focus on functioning is well-matched to adolescents’ needs. Moreover, the inclusion of a transdiagnostic sample aligns with the goal of the novel intervention to address the multifaceted and overlapping nature of these conditions. Drawing on perspectives from adolescents with lived experience of an eating disorder, the design of the digital treatment embedded emotional difficulties and negative self-evaluation as key components [27]. The results of this study show that adolescents’ high levels of difficulties within these domains support the relevance of the treatment in addressing emotional dysregulation, anxiety, depression, and self-worth. This alignment may indicate that the intervention is designed for the users and contexts in which it will be implemented. Furthermore, adolescents’ levels of pretreatment motivation were higher than anticipated when designing the novel intervention. Variability across adolescents highlights that tailored engagement strategies may be an important component for optimizing uptake and adherence to the digital treatment. Although the intervention was developed through a co-design process to reflect the needs of the intended target population, a one-size-fits-all approach may not fully address the heterogeneity among adolescents with eating disorders. Additional flexibility or personalization within the intervention may be needed to better accommodate individual differences and enhance engagement.

### Clinical Implications and Future Directions

Half of the adolescents seeking digital treatment had previously received face-to-face treatment for an eating disorder. This emphasizes the relevance of digital interventions to complement or extend traditional care, particularly for individuals who may not have experienced sufficient benefit from prior treatment. Moreover, the adolescents’ high level of internal pretreatment motivation may suggest that digital treatment can also serve as a viable alternative to face-to-face approaches for some individuals. Suitable options to traditional eating disorder treatment are urgently needed, not only because of existing treatment challenges but also to address future public health care concerns. A remarkable increase in eating disorders among adolescents since the beginning of the COVID-19 pandemic emphasizes a real challenge for health care providers [4,84,85].

Adolescents with anorexia nervosa and bulimia nervosa were excluded from participating. Given the high prevalence of adolescents who go untreated [11], future steps should be taken to offer the intervention to adolescents with these disorders within routine clinical care. A potential solution involves combining digital treatment with periodic in-person medical evaluations. This would also address the needs of those who prefer face-to-face interventions but who also endorse the advantages of digital interventions (eg, availability in times of need and the ability to address stigma associated with help-seeking) [34]. Additionally, digital platforms offer opportunities to integrate tools for monitoring symptoms that may signal medical risk, thereby enhancing patient safety and clinical responsiveness.

Although emotional difficulties are common in adolescents with eating disorders [77-79], these domains are not a primary focus of first-line eating disorder treatments [17]. In the design of mental health technologies, a shift from a top-down approach to a user-driven process necessitates new models of behavior change that move beyond traditional psychotherapy frameworks [76]. The results from this study are in concordance with other studies that address the importance of targeting emotional difficulties in the treatment of eating disorders [29].

The current findings highlight the need to increase diversity among adolescents participating in eating disorder treatment [17,82,83]. In particular, the absence of male participants and the lack of representation of gender-diverse individuals underscore well-documented disparities in help-seeking and access to care. Future research should prioritize recruitment strategies that actively engage these populations, including the use of culturally sensitive language and appropriate measurement tools [17,82,83].

### Limitations

This study has important limitations that need to be addressed. Several aspects of the study design may introduce sampling bias. Recruitment was limited to adolescents eligible for specialist outpatient care within a specific catchment area, which may not reflect the broader geographical population. In addition, this recruitment approach may have constrained the extent to which the sample reflects the broader sociodemographic characteristics and contextual factors considered during the co-design process. The exclusion of those with anorexia nervosa and bulimia nervosa further narrows the sample. In addition, recruitment through self-selection options may favor adolescents who are more motivated, less burdened by barriers to help-seeking, or who already knew that the content would align with their preferences. Moreover, the lack of a control or comparison group may introduce sampling bias, as it is unclear whether participants differ systematically from those who did not participate or from those who would have received other treatments. These factors may reduce the representativeness of the sample and limit generalizability. Another limitation is the lack of systematic reporting and evaluation of the co-design process. Even though it is reasonable to consider that user involvement in the design and development process from the start influences the extent to which adolescents’ characteristics are embedded in the design, we do not know whether it was the involvement of users at a particular phase or the continuous involvement across all phases of design that offers benefits to design research.

### Conclusions

The findings from this study underscore the relevance of digital interventions to complement or extend traditional eating disorder care for adolescents. Furthermore, the findings highlight the importance of enhancing diversity among adolescents participating in digital eating disorder treatments to ensure equitable access and representation. The findings emphasize the importance of designing digital treatments that are sensitive to normative biases and highlight the value of involving a diverse group of adolescents with lived experience of eating disorders in the design and development process from the start. In addition, this study highlights the importance of designing digital treatments that address the multifaceted nature of eating disorders and are tailored to the needs and preferences of a transdiagnostic population. The present findings are important insofar as they may indicate that the key treatment components align with the characteristics of adolescents seeking digital treatment for eating disorders within routine clinical care. Results from the open feasibility trial will show whether adolescents experience the novel digital treatment as acceptable, and credible, and whether the treatment reduces symptoms and increases coping skills.

## Supplementary material

10.2196/93431 Checklist 1 CONSORT checklist.

## Abbreviations

ARFID: avoidant or restrictive food intake disorder
CAMHS: Child and Adolescent Mental Health Services
CIA: Clinical Impairment Assessment Questionnaire
CONSORT: Consolidated Standards of Reporting Trials
DERS-18: Difficulties in Emotion Regulation Scale-18
EDA-5: Eating Disorder Assessment for *DSM-5*
EDE-QS: Eating Disorder Examination—Questionnaire Short
GAD-2: Generalized Anxiety Disorder-2 Scale
*ICD-10*: *International Statistical Classification of Diseases, Tenth Revision*
MINI: Mini-International Neuropsychiatric Interview
PBA: person-based approach
PHQ-2: Patient Health Questionnaire-2
RSES: Rosenberg Self-Esteem Scale
